# Literature Review: Physiological Management for Preventing Postpartum Hemorrhage

**DOI:** 10.3390/healthcare9060658

**Published:** 2021-05-31

**Authors:** Wedad M. Almutairi

**Affiliations:** Maternity and Child Department, Faculty of Nursing, King Abdulaziz University, Jeddah 21589, Saudi Arabia; walmutairi@kau.edu.sa

**Keywords:** skin-to-skin contact, breastfeeding, oxytocin, postpartum hemorrhage

## Abstract

The aim of this paper was to summarize the existing literature regarding postpartum hemorrhage (PPH) and its physiological management (i.e., skin-to-skin contact and breastfeeding). The background surrounding PPH and the role of skin-to-skin contact (SSC) and breastfeeding (BF) in PPH are identified, and these interventions are supported as a crucial means of preventing or minimizing the incidence of PPH. Despite its importance, to the best of my knowledge, an evaluation of this relationship has not yet been undertaken. The narrative literature review approach was used to summarize topic related researches. The search included three databases: CINAHL, PubMed, and Google Scholar. All articles related to the role of SSC and BF in PPH were chosen from the different databases. The findings demonstrate that SSC and BF are cost-effective methods that could be considered practices for the prevention of PPH. Immediate Skin-to-skin contact (SSC) and breastfeeding (BF) are central mediators of the psychophysiological process during the first hour after delivery (the third and fourth stages of labor).

## 1. Introduction

Globally, 295,000 maternal deaths occurred in 2017, resulting in an overall MMR of 211 deaths per 100,000 births. Reducing the maternal mortality rate by 75% is Sustainable Developmental Goal 5 set by the WHO. Obstetric hemorrhage is the leading cause of maternal death worldwide, accounting for 27.1% of all maternal deaths [1]. Of all obstetric hemorrhages, postpartum hemorrhage (PPH) accounts for 72% of these deaths [2]. The active management of the third stage of labor (AMTSL) is a preventive measure for PPH and consists of the administration of exogenous oxytocin (Pitocin), control cord traction, and early cord clamping. However, there is growing evidence of the adverse effects of exogenous oxytocin in normal maternal physiological changes during postpartum and an increased risk of PPH in women who have received higher doses of exogenous oxytocin [3,4,5].

The physiological management of the third stage of labor has received increased attention from researchers. Many studies have been conducted to investigate the effects of early skin-to-skin contact (SSC) between the newborn and the mother and early breastfeeding on the prevention of PPH through their effects on the duration of the third stage of labor and postpartum blood loss [6,7,8,9,10,11,12]. This review aimed to determine the role of SSC and BF on PPH, the duration of the third stage of labor, and the amount of postpartum blood loss, and to clarify the physiological mediator of their effects on women during labor.

## 2. Postpartum Hemorrhage

The definition of postpartum hemorrhage varies across institutions. The WHO defines PPH as “blood loss of 500 mL or more within 24 h after birth, while severe PPH is blood loss of 1000 mL or more within 24 h” [13].The Royal College of Obstetricians and Gynecologists [14] defines PPH as “the loss of 500 mL or more of blood from the genital tract within 24 h of the birth of a baby. Postpartum hemorrhage can be minor (500–1000 mL) or major (more than 1000 mL).” The American College of Obstetricians and Gynecologists (ACOG) defined PPH as blood loss of more than 500 mL for vaginal delivery and more than 1000 mL for cesarean section [15], while their updated definition is “a cumulative blood loss of greater than or equal to 1000 mL or blood loss accompanied by signs or symptoms of hypovolemia within 24 h after the birth process” [16].

Saxton et al. classified the definition of PPH into medical and physiological definitions. Their medical definition of PPH is “blood loss greater than 499 mL,” while the physiological definition is “blood loss of any volume that causes signs of shock or anemia; this volume might vary from woman to woman” (p. 2) [7]. The main cause of PPH is uterine atony, i.e., the failure of the uterus to contract efficiently after placenta delivery, which is known as atonic PPH [17,18,19]. PPH commonly lasts for 24 h, which is called immediate PPH, but there are instances of hemorrhage for up to six weeks after delivery, which is known as delayed PPH [20].

### 2.1. Prevalence and Consequences of PPH

Obstetric hemorrhage is one of the leading contributing factors to maternal deaths worldwide, representing 27.1% of pregnancy-related deaths [1]. This preventable condition has caused challenges to obstetric care globally. Calvert et al. carried out a systematic review and meta-analysis of 71 studies using meta-regression techniques to provide regional estimates of the prevalence of postpartum hemorrhage and found that the overall prevalence rate was 10.8% worldwide for blood loss ≥ 500 cc, with Africa accounting for 25.7% of cases, both Latin America and Asia accounting for 8% each, and 13% in both Europe and North America [21]. The hypermedicalization of the normal birth process may explain the increasing incidence of PPH globally [22,23,24,25]. The incidence of PPH in the United States has increased by 27% from 1995–2004 [26]. A recent cohort study found that the rate increased by 47.5% from 2009–2015 in one tertiary hospital in the United States [17].

Besides contributing to mortality rates, PPH is a source of concern because it is associated with severe maternal morbidity. Grobman et al. estimated the frequency of severe maternal morbidity and assessed the underlying etiologies in the United States, and found that PPH is responsible for almost half of all cases of severe morbidity (46.6%) [27]. Maternal hemorrhage is linked to adverse effects, such as anemia, hypovolemic shock, disseminated intravascular coagulation, acute respiratory distress, renal failure, impaired breastfeeding, and the worsening of existing diseases in mothers, which compromises both physical and psychological health [28,29].

### 2.2. Causes of PPH

The most common cause of PPH is uterine atony [30,31]. The causes of PPH can be summarized by the four Ts, as shown in Table 1: trauma (injuries resulting from lacerations), tone (uterine atony), thrombin (bleeding disorders), and tissue (retained tissues) [31,32,33,34].

### 2.3. Most Common Cause of PPH

The most common factor contributing to the increasing rate of PPH is uterine atony, which is known as atonic PPH. Between 75 and 80% of PPHs result from uterine atony [31,37,41]. This condition develops when the uterine musculature loses its tone. Several factors predispose women to uterine atony, including uterine leiomyomata, multiple gestations, oxytocin augmentation, over-distention of the uterus, chorioamnionitis, prolonged labor, grand multiparity, fetal macrosomia, polyhydramnios, precipitous labor, and exposure to magnesium sulfate regiments and halogenated anesthetics [32].

Despite the knowledge of risk factors, evidence of specific predisposing factors has not yet been documented, and many of the findings are only claims. However, it is agreed that any aspect that affects uterine contraction causes PPH. Since bleeding is a common problem after delivery, predicting cases that will lead to atonic PPH is a challenge. Moreover, atonic PPH can occur without risk factors, and statistics show that atonic PPH occurs more frequently in women without risk factors [25,42]. In this way, all women are equally vulnerable to PPH; thus, preventive measures should be part of each delivery process.

### 2.4. Risk Factors for Atonic PPH

Despite the evidence that PPH occurs independently of the presence of risk factors, the knowledge of them remains the most commonly used approach to predicting the probability of hemorrhage [43]. PPH is a rapid and unpredictable maternal condition that has few leading symptoms. Documented findings have highlighted that 60% of women do not express any sign of increased likelihood of experiencing excessive bleeding [44]. For this reason, PPH remains an idiopathic condition because its cause is not understood. However, in some cases, the PPH victim can express more than one known risk factor. In other instances, PPH is a negligence issue following mismanagement by the health care provider in the third stage of labor. In instances where there is poor service delivery, women can receive poor maternal care that exposes them to complications. The most at-risk group are primigravid mothers because they have a higher rate of PPH with an unknown cause [45].

Some of the risk factors that interfere with the ability of the uterus to contract are the size of the infant (i.e., a large baby weighing more than 4.0 kg); placenta previa; polyhydramnios; exhaustion of muscles following prolonged labor; incomplete separation of the placenta; a full bladder; high parity; weakening of the myometrium, which may fail to contract; multiple gestations; the administration of an exogenous hormone (Pitocin) [3,19,26,33,35,46,47]. One of the negative effects of exogenous oxytocin in normal labor is its reduction in the production of endogenous oxytocin [48,49]. Anemia during pregnancy increases the risk of PPH. The process of clotting is affected, which makes it difficult to stop bleeding and manage PPH, and can lead to death [37].

### 2.5. Prevention of PPH

Two methods are used to manage the third stage of labor to prevent PPH: active and expectant management. Active management involves using early cord clamping, a prophylactic uterotonic, and controlled cord traction to promote the delivery of the placenta. All these operations seek to reduce the risk of hemorrhage. The use of oxytocin reduces the risk of PPH in both cesarean and vaginal births [13,50,51]. During expectant or physiologic management, the placenta is allowed to be delivered spontaneously or with maternal pushing efforts only (hands-off) [9,52].

Many studies have compared the two methods in managing the third stage of labor (active versus expectant), and most have favored active management for preventing severe PPH (>1000 mL of blood loss) [4]. Prediville et al. conducted a trial that compared active and expectant management, and expectant management was associated with a three times higher possibility of PPH compared to the active management group (OR: 3.1, 95% CI: 2.3–4.2) [53]. Rogers et al. conducted a study with randomized subjects who underwent active or expectant management, and actively managed subjects had a 6.8% lower incidence of PPH [54]. However, in the most recent systematic review done by Begley et al., which included 8247 women across seven studies of randomized control and quasi-randomized studies, the findings revealed no significant difference in PPH incidence in low-risk women. In women from the active management group, they found significant increases in maternal diastolic blood pressure, vomiting after birth, after-birth pains, use of analgesia from birth to discharge from the labor ward, and more women returning to the hospital with bleeding. A decrease in the baby’s birth weight was also related to active management, reflecting the lower blood volume from interference with placental transfusion [55].

Hence, it can be concluded that there is still uncertainty surrounding the optimal management of PPH. Some researchers suggested that physiological management might be an optimal way to help mothers release oxytocin, which results in the efficient separation of the placenta without introducing possible complications through medical interventions, especially in low-risk women. Recently, there has been a call to decrease nonessential interventions for healthy women in normal labor due to the increasing rate of PPH incidence in developed countries, such as the United States [3,17,56]. Therefore, introducing skin-to-skin contact (SSC) and immediate breastfeeding (BF) to prevent PPH are considered innovative and crucial ways for the optimal management of PPH [8,9,49].

### 2.6. Significance of a Review about Prevention Measures for Atonic PPH

Seeking a preventive treatment for PPH is important because the prevalence rate has remained unchanged with current treatments. The incidence of atonic PPH is greater in low-income, third-world populations that do not have access to medication to stop hemorrhages. Atonic PPH is also a growing problem in developed countries such as the United States, where the rate of PPH has doubled over the last 10 years [19,57,58]. Endogenous oxytocin does not have the adverse effects that exogenous oxytocin has, and the need for a readily available, low-cost treatment is great [59,60]. Physiological management, such as SSC and BF, can be utilized to promote oxytocin production, which may ultimately contribute to a reduction in or the prevention of PPH [8,49,61].

## 3. Oxytocin

Oxytocin is a peptide hormone that occurs naturally in all animals, and it affects the brain and peripheral systems during and after birth. It is produced in the magnocellular nuclei of the supraptic nucleus (SON) and paraventicular nucleus (PVN) of the hypothalamus, and it accumulates and is released into the blood circulation from the posterior pituitary gland in response to different types of stimuli, such as suckling during breastfeeding, labor, and various types of non-noxious stimuli, including touch, warmth, and stroking of all parts of the body [62,63]. In addition to acting as a hormone in the peripheral system, oxytocin also acts as a neurotransmitter in the brain. Oxytocin release is controlled by a positive feedback system. The most important role of oxytocin is to bind oxytocin receptors in the uterus and stimulate uterine contractions to expel pregnancy products from the uterus and close the spiral blood vessels in the placental site to stop bleeding [64]. In preparation for delivery, estrogen helps to increase the number and sensitivity of oxytocin receptors. Oxytocin also plays an important role in lactation after birth, causing the mammary glands to contract and eject milk. Hence, the woman’s body is naturally prepared for the effects of oxytocin during labor and birth. Ultimately, oxytocin can be considered as the fuel for the uterus to contract effectively during labor and strengthen the uterine tone. Early interventions, such as SSC and BF, are recommended. Many studies have evaluated the time of the maximum elevation of oxytocin during and after birth, and it has been shown that the most sensitive time is when a woman’s oxytocin level peaks, during the first 60 min after birth. Researchers have attempted to maximize this elevation in oxytocin, and they measured oxytocin levels during this sensitive period regarding mother–infant SSC. They found that oxytocin levels significantly increased in comparison with normal elevation rates 15, 30, and 45 min after birth in women who experienced SSC [63,65].

Studies have found many psychophysiological effects that are associated with oxytocin, including a sense of calm and connection between mother and baby during BF for short- and long-term adaptation, anxiolytic effects, lower blood pressure, an elevated pain threshold, a decrease in plasma cortisol, and antidepressant effects [65,66].

Some studies that found a direct relationship between SSC and the level of oxytocin include Feldman et al. who focused on the level of oxytocin in saliva and plasma in the parent, [67] and Gordon et al., who investigated OXT levels in fathers and mothers when touching their baby [68]. Other researchers have studied the relationship between BF and oxytocin levels: Uvnäs-Moberg thoroughly researched the connections between oxytocin and BF, and between oxytocin and SSC [63,69]. The research found that SSC and BF both significantly increase the level of maternal oxytocin.

## 4. Physiological Management to Prevent or Minimize the PPH Incidence Rate

PPH can be managed by employing nonpharmacological treatments. One of these approaches is to apply nipple stimulation during labor. This concept has been documented in the literature since the 1800s and it involves massaging the breasts [70,71,72]. Nipple stimulation affects the pituitary gland, which leads to the release of oxytocin, a hormone that is associated with inducing labor. This intervention is carried out within 15 min after delivery and seeks to achieve a short-term burst of oxytocin. Spiking levels of the hormone lead to an increase in uterine contractions. The increased uterine contractions lead to the expulsion of the placenta and all materials, such as blood clots, thus preventing PPH [71,72,73].

One of the most important studies investigating the effects of BF on uterine tone was conducted by Chua et al. [74]. It included 11 subjects that were divided into two groups: immediate BF was the treatment in group A and nipple stimulation (manually by mothers) was the treatment in group B. Prophylactic oxytocin was not given in the third and fourth stages of labor and a transducer-tipped catheter was inserted into the uterine cavity to record the postpartum uterine activity. The findings revealed a 17−730% increase in uterine activity with breastfeeding and nipple stimulation when compared with the averages found pre- and post-intervention. All subjects demonstrated increases in their uterine activity, with the major increase among women who had breastfed their babies compared to women who were manually stimulating their nipples. The median increase in uterine activity with breastfeeding was 93%, and the median increase in uterine activity with synthetic oxytocin (the preferred drug used to prevent PPH) was 96.5% [74]. Therefore, natural measures have value in the prevention of PPH and may avoid unnecessary intervention during the normal processes of labor using high-alert medications, such as Pitocin [25,40,41,42,43,44,45,46,47,48,49].

Another physiological measure that influences oxytocin levels positively is SSC, which is a multisensory approach. SSC stimulates the pituitary gland to release oxytocin). Elevated levels of the hormone in the blood, with the uterus being the target organ, lead to increased contractions, thus preventing PPH. This approach is relatively new, and scholars have not yet explored this intervention extensively [8,49,65].

## 5. Skin-to-Skin Contact (SSC)

SSC is defined as the process of holding a diaper-clad infant in an upright position on the chest of the mother while at the same time maintaining skin-to-skin contact. The infant must be covered to maintain an optimal temperature and prevent respiratory ailments [75,76]. Saxton, Fahy, and Hastie described the process of SSC as follows:

The naked healthy newborn baby is placed on the mother’s bare abdomen/chest (depending upon cord length) immediately after birth. In this position, the baby has ready access to the maternal nipple and can hear the mother’s heart. The mother and the baby should be covered with a warmed blanket and left there undisturbed for at least an hour. The mother and baby are carefully and unobtrusively observed to ensure optimal adaptation of both following birth [7].

However, this process should not only be employed with mature infants but also with medically stable, premature infants because of the beneficial effects of the intervention. The approach has beneficial maternal outcomes, including improved maternal–infant bonding, promotion of milk production, increased success with breastfeeding, enhanced parental psychological and emotional wellbeing, decreased clinical depression, decreased placental expulsion time, assistance with uterine involution, decreased maternal anxiety, increased maternal satisfaction, reduced pain during the episiotomy, increased hematocrit levels, reduction in lochia, shorter maternal hospital stay, and decreased maternal cortisol levels. In summary, maternal physiology and psychology benefit from SSC in many ways [77].

### 5.1. SSC Physiology and Its Relation to Uterine Atony

SSC is a multisensory method that stimulates pituitary glands and promotes the production of endogenous oxytocin. Holding, touching, smelling, and/or seeing a baby, and even hearing baby sounds, accelerates the speed with which social impulses are sent to the brain, which has an effect on the production of oxytocin, the social hormone. This concept relates to all types of SSC, but in kangaroo mother care, oxytocin produced helps to promote uterine contractility [7,62]. Therefore, SSC is one of the interventions that stimulate the release of oxytocin, helping with uterine contractility, which prevents uterine atony, the main cause of PPH.

### 5.2. Previous Studies of the Relationship between SSC and Uterine Atony

According to Moore et al., SCC reduces the chance of uterine atony occurring. In their review, the researchers criticized the trend in Western culture to separate the mother and infant. Instead, they encouraged early SSC, including placing the infants on the bare chest of their mothers. They claimed that SSC has proven neuroscience advantages that are associated with inducing neurobehaviors and ensuring the fulfillment of mothers’ biological needs [78].

In a study by Handlin et al., the researchers revealed that SCC decreases cortisol and ACTH levels in the blood. The direct response to this change was an increase in endogenous oxytocin levels, which was inversely correlated with the level of the two hormones. Their findings discovered a significant positive correlation between the duration of SSC and endogenous oxytocin levels and the negative correlation with the cortisol level; a longer SSC duration depressed cortisol levels. Moreover, from this study, it seems that using exogenous oxytocin has an adverse effect on the level of endogenous oxytocin and the cortisol level increased in the group who received exogenous oxytocin [65,79].

In the study conducted by Dordevic, 216 mothers spent time experiencing SSC with their babies after delivery (study group) and a control group of 216 mothers did not have SSC with their babies. Lochia measured the uterine involution, postdelivery bleeding on the third day after birth, sanitary napkin consumption, and the hospitalization length. The findings revealed that the SSC group experienced fewer instances of severe uterus involution; less postdelivery anemia, as measured by hemoglobin values and the number of erythrocytes; less sanitary napkin consumption; a shorter hospital stay [80].

Matthiesen et al. investigated the effects of an infant hand massage and suckling on maternal oxytocin. The researchers video-recorded every movement by infants from birth until the first breastfeeding, and maternal blood samples were collected every 15 min to analyze the oxytocin levels via a radioimmunoassay. The results indicated that even infant hand massage increases the level of maternal oxytocin, which has a significant effect on uterine contraction and milk ejection [62].

## 6. Breastfeeding (BF)

Breastfeeding is the act of feeding an infant with breast milk directly from the breast by the mother. According to the WHO [42], this approach should be the only way of meeting infants’ nutritional needs during the first six months of life, after which time, complementary food can be introduced. The feeding should be initiated within one hour following the baby’s delivery, as the act not only provides the child with a chance to suckle the rich colostrum, but also stimulates oxytocin, which helps to relieve the mother’s stress from the delivery process. Another definition is “any attempt by the baby to suckle the breast within 30 min after the birth” [7]. Thus, immediately initiating BF is critical, and it should be emphasized that researchers found early SSC to accomplish the goal of immediate and exclusive BF.

A randomized control trial conducted by Moore and Anderson identified the effects of early SSC on BF. The study included 21 women who met the study criteria, and the researchers used the infant breastfeeding assessment tool (IBAT) and the index of breastfeeding status (IBS) to measure the effects. Their findings revealed that the SSC group had a higher rate of suckling competency during the first breastfeeding (*p* < 0.02) and were able to effectively breastfeed sooner (*p* < 0.04). Consequently, very early SSC enhances breastfeeding success in the first 2 h postpartum [81].

### 6.1. Breastfeeding Physiology and Relation to Uterine Atony

Just like nipple stimulation, breastfeeding aids in achieving an increase of oxytocin levels in the blood. The rise in oxytocin leads to an increase in contraction of the myometrium, helping to expel all pregnancy products. The contractions also help to prevent excessive bleeding because they help to close blood vessels [7].

### 6.2. Previous Studies on the Relationship Between Breastfeeding and Uterine Atony

According to the findings of previous studies, BF is part of the management practices that are encouraged during the fourth stage of labor. This stage is initiated by the delivery of the placenta and lasts for 4 h. BF plays a major role in creating a parent–infant bond and preventing PPH. Geller et al. recommended BF as one of the approaches that should be utilized in developing nations. In their study, they argued that global maternal mortality has remained constant over the last 10 years despite the efforts of international agencies to change this. They highlighted the effectiveness of pharmaceutical and surgical interventions that have been developed. However, they concluded that these approaches are not feasible or practical in poor settings where PPH is rampant because of home delivery. They proposed cheap nonpharmacological interventions, such as BF, which are effective and easy to apply in any environment [82].

## 7. Previous Studies on the Effects of Breastfeeding and Skin-to-Skin Contact on PPH

The greatest risk during the postpartum period is PPH, which can be prevented by boosting oxytocin hormone levels through physiological measures, such as BF and SSC.

Saxton et al.’s study on the effects of SSC and BF at birth on the incidence of PPH was a retrospective cohort study that aimed to determine whether immediate SSC and BF affected the rate of PPH. The database included 10,000 women who were analyzed for incidences of PPH. The findings revealed statistically significant results showing that women who breastfeed or experience SSC are less likely to have PPH (*p* < 0.0001 for both SSC and BF) [83].

Bingham found that the use of Pitocin made PPH persistent. As such, she called for the use of nonpharmacological interventions, which she argued are not only effective but also promote the mothers’ health. She found that the use of Pitocin leads to a decrease in a woman’s HCT and HGB by 24.0 and 8.5 after delivery, respectively, with a further decrease to 22.3 and 7.9 at 48 h after delivery, respectively [58]. These findings showed that the pharmacological approach should be discouraged and replaced with SCC and BF as interventions to prevent PPH, which are consistent with Campbell-Yeo et al.’s findings [7,58,59,60,61,62,63,64,65,66,67,68,69,70,71,72,73,74,75,76,77,78,79,80,81,82,83,84].

A retrospective cohort study including 154 cases of atonic PPH found that women who were diagnosed with atonic PPH and had SSC and BF bled less compared to women who were diagnosed with PPH but did not have SSC and BF during their first hour after giving birth [17].

## 8. Previous Studies that Investigated the Effects of BF and SSC on the Duration of the Third Stage of Labor and Postpartum Blood Loss

Regarding SSC, a quasi-experimental study that was conducted to examine the effects of SSC on the duration of the third stage of labor that included 108 women found that the SSC group experienced a shorter duration of the third stage of labor (6 ± 1.7 min, *p* < 0.01) [11]. A recent systematic review and meta-analysis conducted by Karimi et al. included six studies and found that the SSC group had a shorter duration of the third stage of labor compared to the control group, with a mean difference of −1.33 min (95% CI: 0.36–2.31, *p* < 0.01) [10].

With regard to BF, a quasi-experimental study was conducted to examine the effects of BF on uterine consistency and postpartum blood loss, which included 100 women. The results revealed that the BF group experienced significantly less blood loss (mean: 194 mL) than the control group (194 vs. 260 mL, *z* = 3.226, *p* < 0.01) [70]. Both SSC and BF have an influence on the amount of blood loss and the duration of the third stage of labor.

## 9. Conclusions

Uterine atony is the main cause of PPH, accounting for 70% of PPH cases. However, the condition is highly responsive to physiological management approaches, such as BF and SSC. Both SSC and BF have an effect on endogenous oxytocin and reduce the production of stress hormones in women, which interferes with the action of oxytocin that stimulates uterine contractility and efficiently prevents atonic PPH. Furthermore, SSC and BF have evident effects on the duration of the third stage of labor and postpartum blood loss. The findings of this review demonstrate that SSC and BF are cost-effective methods that could be listed in practices of PPH prevention for optimal maternal care and PPH management because immediate SSC and BF are central mediators of the psychophysiological process during the first hour after delivery.

## Figures and Tables

**Table 1:** Causes of postpartum hemorrhage.

Ts	Causes
Trauma	Genital tract trauma: episiotomy; forceps delivery; cervical, vaginal, or perineal lacerations; ruptured uterus [32]
Tone	Uterine atony due to the induction of labor, oxytocin use, prolonged labor, anesthesia, uterine overdistension (multiple pregnancies or polyhydramnios or large fetus) [35,36,37,38]
Tissue	Placenta previa and placenta accreta [32,39,40]
Thrombin	Coagulation disorders: disseminated intravascular coagulopathy; liver dysfunctions; thrombocytopenia; inherited bleeding dysfunction, such as von Willebrand diseases; anticoagulant therapy [32,34].
